# Molecular Insights into Defense Responses of Vietnamese Maize Varieties to *Fusarium verticillioides* Isolates

**DOI:** 10.3390/jof7090724

**Published:** 2021-09-04

**Authors:** Trang Minh Tran, Maarten Ameye, Sofie Landschoot, Frank Devlieghere, Sarah De Saeger, Mia Eeckhout, Kris Audenaert

**Affiliations:** 1 Laboratory of Applied Mycology and Phenomics, Department of Plants and Crops, Faculty Bioscience Engineering, Ghent University, 9000 Ghent, Belgium; Maarten.Ameye@UGent.be (M.A.); Sofie.Landschoot@UGent.be (S.L.); 2 Laboratory of Applied Mycology, Department of Food Technology, Safety and Health, Faculty of Bioscience Engineering, Ghent University, 9000 Ghent, Belgium; Mia.Eeckhout@UGent.be; 3 Research Unit Food Microbiology and Food Preservation, Department of Food Technology, Safety and Health, Faculty of Bioscience Engineering, Ghent University, 9000 Ghent, Belgium; Frank.Devlieghere@UGent.be; 4 Center of Excellence in Mycotoxicology and Public Health, Department of Bioanalysis, Faculty of Pharmaceutical Sciences, Ghent University, 9000 Ghent, Belgium; Sarah.DeSaeger@UGent.be; 5 Research Unit of Cereal and Feed Technology, Department of Food Technology, Safety and Health, Faculty of Bioscience Engineering, Ghent University, 9000 Ghent, Belgium

**Keywords:** benzoxazinoids, *Fusarium verticillioides*, *Fusarium* ear rot, maize, mycotoxins, phytohormones, Vietnam

## Abstract

*Fusarium* ear rot (FER) caused by *Fusarium verticillioides* is one of the main fungal diseases in maize worldwide. To develop a pathogen-tailored FER resistant maize line for local implementation, insights into the virulence variability of a residing *F. verticillioides* population are crucial for developing customized maize varieties, but remain unexplored. Moreover, little information is currently available on the involvement of the archetypal defense pathways in the *F. verticillioides*–maize interaction using local isolates and germplasm, respectively. Therefore, this study aims to fill these knowledge gaps. We used a collection of 12 *F. verticillioides* isolates randomly gathered from diseased maize fields in the Vietnamese central highlands. To assess the plant’s defense responses against the pathogens, two of the most important maize hybrid genotypes grown in this agro-ecological zone, lines CP888 and *Bt/GT* NK7328, were used. Based on two assays, a germination and an *in-planta* assay, we found that line CP888 was more susceptible to the *F. verticillioides* isolates when compared to line *Bt/GT* NK7328. Using the most aggressive isolate, we monitored disease severity and gene expression profiles related to biosynthesis pathways of salicylic acid (SA), jasmonic acid (JA), abscisic acid (ABA), benzoxazinoids (BXs), and pathogenesis-related proteins (PRs). As a result, a stronger induction of SA, JA, ABA, BXs, and PRs synthesizing genes might be linked to the higher resistance of line *Bt/GT* NK7328 compared to the susceptible line CP888. All these findings could supply valuable knowledge in the selection of suitable FER resistant lines against the local *F. verticllioides* population and in the development of new FER resistant germplasms.

## 1. Introduction

Contamination of maize by mycotoxins is a global issue threatening food safety and security, especially in the tropics. Mycotoxins are secondary metabolites produced by filamentous fungi such as *Fusarium verticillioides* and *Aspergillus flavus* that often occur and colonize crops in the field or during storage [1]. Due to the acute toxicity of mycotoxins, legislated regulations for the acceptable limits for each mycotoxin in food and feed have been established in many countries [2]. To tackle this problem, studies in breeding for resistance to mycotoxigenic fungi have become an attractive topic in the community of plant breeders.

*Fusarium verticillioides* is the primary causal agent of *Fusarium* ear rot (FER) in most tropical maize-growing areas [3,4,5]. This pathogen is capable of producing mycotoxins, primarily fumonisins (incl. fumonisin B_1_ (FB_1_), FB_2_ and FB_3_), resulting in low yield and grain quality as a consequence of the FER disease [6]. From a toxicological standpoint, FB_1_ is classified into group 2B as being possibly carcinogenic to humans [7]. The chemical structure of FB_1_ is similar to sphingoid bases such as sphinganine and sphingosine. Based on this structural similarity, FB_1_ has the ability to disrupt the sphingolipid biosynthesis pathway in a mammalian cells, resulting in carcinogenesis [8,9]. In maize plants, Glenn et al. (2008) [10] and Zitomer et al. (2010) [11] have demonstrated that FB_1_ produced by *F. verticillioides* is a virulent factor, whereas Desjardins and Plattner (2000) [12] showed that this metabolite is not involved in the pathogenesis of *F. verticillioides*. In Vietnam, *F. verticillioides* has been frequently discovered in the central highlands’ maize fields between 2017 and 2019 [4]. In particular, contamination of maize with fumonisins from the field to the postharvest chain was documented in this agro-ecological zone [4,13]. An evaluation of virulence variability of the local *F. verticillioides* population is needed as a first step in developing locally adapted resistant cultivars.

Breeding for resistance to FER is the most eco-friendly, economical and sustainable strategy, and has been attracting the interest of numerous plant breeders [14,15]. However, to date, no commercial hybrid maize lines with complete FER resistance are available, although transgenic *Bt* insect-resistant lines (*Bt* genes derived from *Bacillus thuringiensis* encoding insecticidal proteins) have been suggested as effective FER control measures [16].

In recent years, although the *F. verticillioides*–maize interaction has been gradually uncovered, it is still not completely understood due to its multi-layered nature. Insights into the transcriptional levels of plants and the identification of defense-mediated hallmark genes upon infection by *F. verticillioides* can help to improve understanding of this intricate interaction, and may be helpful for the development of FER resistant host lines. Phytohormones e.g., salicylic acid (SA), jasmonic acid (JA), and abscisic acid (ABA) play a pivotal role in the defense response of maize against *F. verticillioides* [14,17,18]. On the other hand, the induction of hallmark genes associated with the benzoxazinoids (BXs) (i.e., an antimicrobial and insecticidal secondary compound in maize) biosynthesis pathway has been demonstrated linked to plant defense mechanisms against the pathogens [19,20]. The involvement of genes encoding pathogenesis-related proteins (PRs), especially β-1,3-glucanase, chitinase and pathogenesis-related protein 10 (PR10 protein), has also been observed in the defense responses of maize plants against *F. verticillioides* [17,21].

In the present work, we aim to evaluate the level of resistance or susceptibility of Vietnamese maize genotypes to the local *F. verticillioides* field isolates. To achieve this, we used a set of 12 local isolates of *F. verticillioides* derived from our previous study [4] together with the two most important hybrid maize cultivars grown in the central highlands of Vietnam, lines CP888 and *Bt/GT* NK7328. We examined the transcriptional regulation of genes involved in the biosynthesis of phytohormones (e.g., SA, JA, and ABA), BXs, and pathogenesis-related proteins (e.g., PR10 protein, glucanase and chitinase) within each cultivar upon *F. verticillioides* root infection. This work is timely and important for the proposal of tailored control strategies by introducing local resistant maize genotypes and biocontrol agents against the residing *F. verticillioides* population.

## 2. Materials and Methods

### 2.1. Preparation of Fusarium verticillioides Isolates

Twelve *F. verticillioides* isolates were randomly isolated from maize fields in the central highlands of Vietnam (Table S1). This *F. verticillioides* population consisted of isolates derived from maize fields during three different crop seasons: the autumn-winter crop of 2017, the summer-autumn crop of 2018, and the autumn-winter crop of 2018, in two locations, Dak Lak and Dak Nong [4].

For the preparation of spore suspensions, each isolate was cultured on a PDA (Potato Dextrose Agar, 40 g L^−1^) (Sigma Aldrich, Overijse, Belgium) plate for 7 days at 25 °C, prior to transferal into a cabinet equipped with near-UV lights (12 h light/12 h darkness) for 7 days to induce sporulation. A spore suspension of each isolate was obtained by rubbing the mycelial surface with 20 mL of sterile water and filtering through an autoclaved Mira-cloth filter into a 50 mL sterile tube. A final concentration of each spore suspension 10^7^ conidia mL^−1^ was made based on microscopic counts using a Bürker chamber. All isolates were maintained in spore suspension with 50% glycerol at −80 °C for a long-term period.

### 2.2. Multilocus DNA Sequence Based Identification of Fusarium verticillioides

Total fungal DNA extraction was conducted following [4]. Three different fungal gene regions consisting of translation elongation factor 1α (*EF-1α*) and the largest subunits of RNA polymerase (*RPB1* & *RPB2*) were amplified (Table S2). All PCR reactions were performed using the GeneAmp PCR system 97,000 PCR (Applied Biosystem, Foster City, CA, USA) following the protocol of Tran et al. 2021 [4]. Amplicons were separated on a 1.5% (*w*/*v*) agarose gel, stained with ethidium bromide for 30 min and then visualized using the Molecular Imager^®^ Gel Doc^TM^ XR+ System with Image Lab^TM^ Software (BIO-RAD, Hercules, CA, USA). PCR products were purified using the E.Z.N.A.^®^ Cycle-Pure Kit (VWR International, Leuven, Belgium) before sending to LGC Genomics (LGC group, Berlin, Germany) for Sanger sequencing.

### 2.3. Pathogenic Assay of Fusarium Verticillioides in Two Inbred Maize Lines

#### 2.3.1. Seed Germination Assay

The line CP888 (originating from C.P. group, Vietnam) and the insect-resistant transgenic line *Bt/GT* NK7328 (originating from Syngenta group, Vietnam) are two important maize lines grown in Vietnam’s central highlands [4]. In each treatment, thirty seeds were disinfected by soaking in 30 mL of 1% sodium hypochlorite (NaOCl) for 30 s, followed by washing with sterile water five times, and finally drying in a biosafety cabinet. The seeds were then soaked in 25 mL of each *F. verticillioides* spore suspension (10^7^ conidia mL^−1^) for 1 h, and transferred into a sterile petri plate containing a Whatman paper moistened with 4 mL sterile water (10 seeds per plate), and inoculated for 7 days at 25 °C. Sterile water was used as a control. Germination rate and lengths of roots and shoots were used to evaluate the susceptibility of these two maize lines to *F. verticillioides* isolates.

#### 2.3.2. *In-Planta* Assay

In each treatment, five maize seeds were disinfected with 1% NaOCl and transferred to a rectangular plastic pot (20 × 16 × 6 cm) with vermiculite substrate (Vermex, Soprema, Belgium) to germinate for 5 days. The five germinated seeds were soaked in a fungal suspension (10^7^ spores mL^−1^) for 1 h prior to transferal to five glass tubes containing mixed sand (200 g/tube) (Figure S1). Five days before planting, 3 g polymer gel (DCM Aquaperla, Grobbendonk, Belgium) absorbed in 300 mL tap water was mixed with 2 kg non-sterile fine sand (Vosschemie Benelux, Lier, Belgium) [22]. Every two days, 10 mL of tap water was applied. After 2 weeks of planting, the maize plants were harvested. Based on the length of leaves and roots, and the fresh biomass of leaves and roots, the susceptibility of plants to pathogens was evaluated. Five control plants were treated with sterile water. For the time-series assay, the following time points were chosen: 1 day after infection (dai), 2 dai, 4 dai, and 8 dai. Five plants from each treatment were harvested at each time point. Length and fresh biomass of root and leaf were measured and then stored at −20 °C for RNA extraction.

### 2.4. RNA Extraction and RT-qPCR

Roots and leaves were crushed with liquid nitrogen prior to RNA extraction, using TRizol reagent (Sigma Aldrich, Belgium) according to the manufacturer’s instructions. RNA concentration was determined using a Quantus fluorometer (Promega, the Netherlands). cDNA of each RNA template was synthesized using the iScript^TM^ kit (Bio-rad, Belgium) and diluted 5 times with nuclease-free water. RT-qPCR assay was performed using a CFX96 Tough Real-time PCR Detection System (Bio-rad, Belgium). Each PCR reaction contained 6.25 µL Gotag^®^qPCR master mix (Promega, The Netherlands), 2 µL cDNA, 0.625 µL each primer (5µM), and 0.208 µL CXR dye (Promega, The Netherlands), and 2.292 µL nuclease-free water. The thermal programme was set up as follows: 95 °C for 3 min; 39 cycles of 95 °C for 10 s, and 60 °C for 30 s, followed by a melting curve acquisition from 65 to 95 °C with the rate of 0.5 °C/s. The primers used for all genes are shown in Table S3. Elongation factor 1α (*EF-1α*) and β tubulin (*β-TUB*) primers were used as reference genes. Gene expression analysis was done using qBase^+^ software (Biogazelle, Belgium). Fold change was calculated by diving the CNRQ values (calibrated normalized relative quantities) of the infected samples by values of the control samples.

### 2.5. Statistical Analysis

All heat maps and boxplots were generated using the R software v.4.0.2 with the packages ggplot2 and gplots (https://cran.r-project.org/ (accessed on 15 July 2021)). A one-way ANOVA test followed by a post-hoc Tukey’s test was used in cases of normal distribution, otherwise, a non-parametric Kruskal-Wallis test followed by a post-hoc Dunn’s test -test was applied. All analyses were tested at a significance level of α = 0.05.

## 3. Results

### 3.1. Susceptibility of Two Maize Genotypes to Vietnamese Maize Fields’ Fusarium verticillioides Population on Germination Seeds

**Figure 1:**
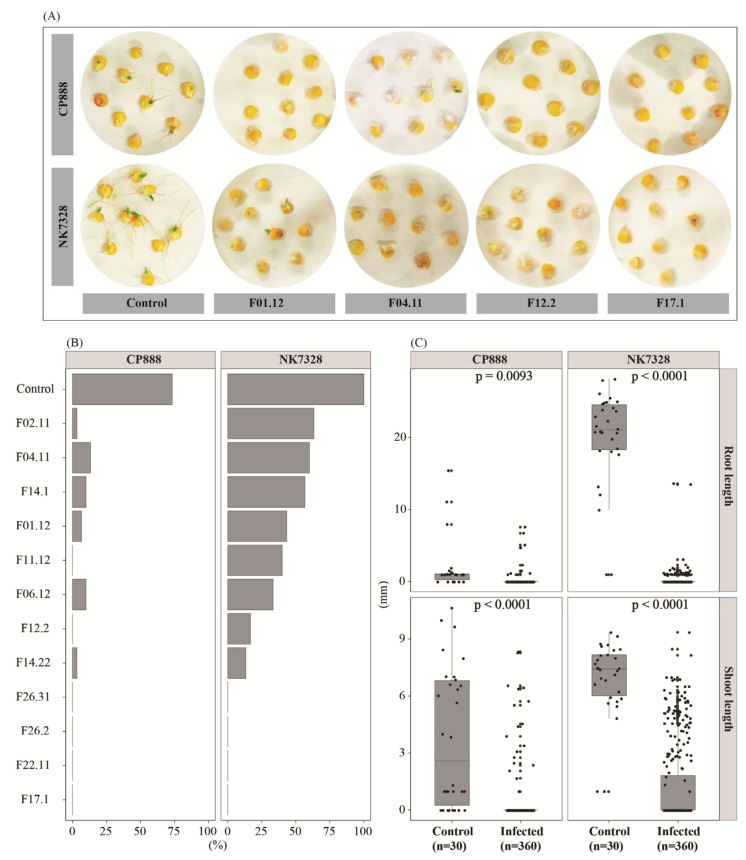
(**A**) Germination of seeds on moistened Whatman papers by treatments. (**B**) Germination rates (%) of CP888 and *Bt/GT* NK7328 seeds when infected with each *Fusarium verticillioides* isolate at 7 days after inoculation. Thirty seeds were infected with each isolate at a concentration of 10^7^ conidia mL^−1^, while sterile water was utilized for a control treatment. (**C**) Impacts on root length and shoot length upon infection by pathogens. Statistical differences were interpreted using a Student’s *t*-test at a significant value of α = 0.05.

In this work, we used the two main growing maize lines, CP888 and *Bt/GT* NK7328, in the central highlands of Vietnam. The susceptibility of these two cultivars to locally isolated *F. verticillioides* isolates was assessed by using a seed germination assay within 7 days. Data show that line CP888 was more susceptible to *F. verticillioides* than line NK7328 (Figure 1). Evidently, when infected with the pathogens, a significantly lower median germination rate was observed in line CP888 seeds (*n* = 360, 3 ± 6%) when compared to line NK7328 seeds (*n* = 360, 33 ± 9%) (one sided *p* = 0.03, Kruskal-Wallis test) (Figure 1B). The transparent pathogenic phenotypes of germination seeds upon infection by the isolate F01.12, at which the seed germination rate of line CP888 (*n* = 30) was 7% versus 43% for line NK7328 (*n* = 30) (Figure 1A), made it clear that the germination of both cultivars was strongly impacted by the infection by isolates F17.1, F22.11, F26.2, and F26.31.

In terms of the impacts on roots and shoots, in line CP888, shoot length was significantly reduced when infected with the pathogens, compared to the control CP888 seeds (Figure 1C). The median shoot length was 0 ± 0.1 mm (in the infected seeds) vs. 2.6 ± 0.7 mm (in the control seeds) (*p* < 0.0001). Similar results were obtained for line NK7328. Likewise, a significant reduction of root length was observed in both cultivars (Figure 1C).

In conclusion, the germination assay-based data show that the germination of both cultivars was significantly impacted by the infection of *F. verticillioides*. By measuring the root and shoot length, a higher disease level from the infection was observed in seedlings of line CP888 compared to seedlings of line NK7328.

### 3.2. Susceptibility of Two Maize Genotypes to Vietnamese Maize Fields’ Fusarium verticillioides Population on Maize Seedlings

**Figure 2:**
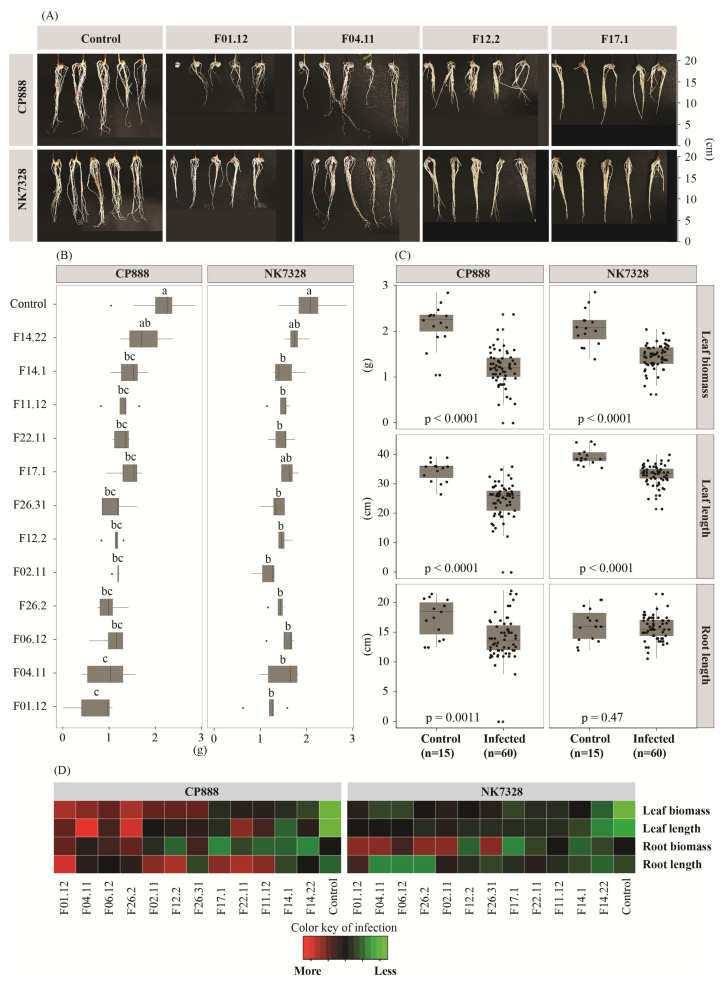
(**A**) Phenotype of roots by treatment upon 14 days. (**B**) Impact on leaf biomass upon infection with each *F. verticillioides* isolate. Two hybrid maize cultivars CP888 and *Bt/GT* NK7328 were used. Different letters above each boxplot indicate significant difference within cultivar using a parametric One-way ANOVA test followed by a post-hoc Tukey’s test at a significance level of α = 0.05. (**C**) Impacts on leaf biomass, leaf length and root length. A Student’s *t*-test was done. (**D**) A heat map of combined growth indicators by isolates. More red is more aggressive to pathogens.

Here, we evaluated the susceptibility of two maize genotypes when infected with the pathogens on maize seedlings within 14 days. Upon seedling infection by *F. verticillioides*, for line CP888, a clear infection was confirmed through root phenotypes (Figure 2A). Notably, root length was significantly reduced, by 14.0 ± 3.6 cm (in the infected seedlings) vs. 17.5 ± 3.2 cm (in the control seedlings) (*p* = 0.0011) (Figure 2C). The infection also resulted in a significant reduction of leaf length and leaf biomass in the seedlings of line CP888. In contrast, a lower disease level was observed at the roots of line NK7328 (Figure 2A,C). There was no significant difference in root length compared to the control seedlings (*p* = 0.47). However, a significant reduction was observed in leaf length and leaf biomass in response to the infection. (Figure 2C). A heat map also indicated that line CP888 was more susceptible to the pathogens when compared to line NK7328 (Figure 2D).

On the other hand, data show a high virulence variability of *F. verticillioides* isolates upon infection in maize seedlings (Figure 2B). In fact, based on the effect on leaf biomass of line CP888 seedlings, a varying virulence level amidst 12 isolates was indicated (Figure 2B). For example, isolate F01.12 showed a high virulent level on both maize seedlings, while a non-virulent level was observed in isolate F14.22. Higher virulence was observed in isolates F01.2, F04.11, F06.12, F26.2, F02.11, and F26.31. By contrast, F14.22, F14.1, and F11.11 were less virulent.

In conclusion, the *in-planta* data show that higher infection levels were observed in line CP888 than in line *Bt/GT* NK7328 upon infection with *F. verticillioides*. We can conclude that line CP888 maize is more susceptible to *F. verticillioides* than line *Bt/GT* NK7328. In addition, this *F. verticillioides* population was shown to have highly variable virulence.

### 3.3. Differential Resistance Responses between Susceptible and Resistant Cultivars upon Infection by Fusarium verticillioides on Maize Seedlings

**Figure 3:**
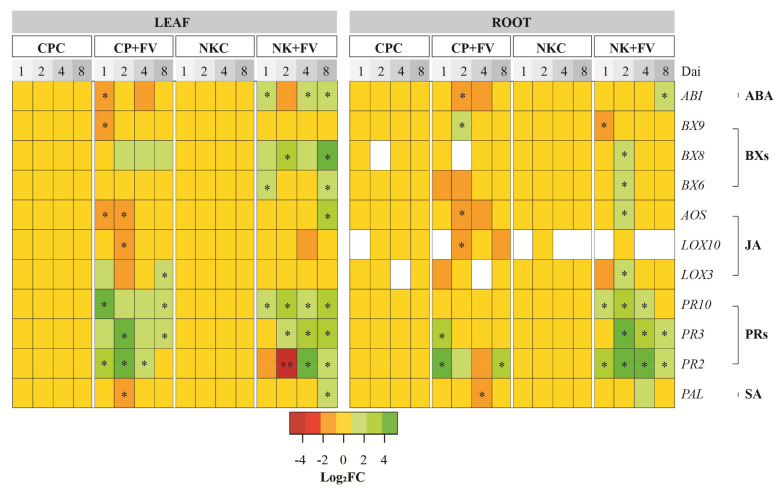
Heat map of the differential gene expression levels in leaf and root of two hybrid maize lines, CP888 and *Bt/GT* NK7328. The colors indicate the fold changes (FC) of gene expression in log base 2. Up-regulation of each gene is shown in green (increased abundance), down-regulation is shown in red (decreased abundance), no change is shown in yellow, and no expression is indicated in white. *Fusarium verticillioides* (FV); salicylic acid (SA); jasmonic acid (JA); benzoxazinoids (BXs); abscisic acid (ABA). Dai = days after inoculation. Defense response was monitored at 1, 2, 4 and 8 dai. Control CP888- or NK7328 plants mock-infected with sterile water (CPC and NKC, respectively). CP888- or NK7328 plants infected with *F. verticillioides* strain F01.12 (CP+FV and NK+FV, respectively). Symbols (*) and (**) depict significant difference at significance levels of α = 0.05 and α = 0.01, respectively, between the infected and the control mock plants within a cultivar using one-tailed Kruskal-Wallis tests.

The aforementioned data showed that maize line CP888 was more susceptible to *F. verticillioides* than maize line *Bt/GT* NK7328 maize. Hence, in this work, to further understand the mode of action of *F. verticillioides* on the resistance mechanism of these two maize cultivars, we used the highly virulent isolate F01.12, and then assessed the transcript levels of 11 genes associated with regulation of SA, JA, ABA, BXs, and PRs biosynthesis. Data are shown in Figure 3.

#### 3.3.1. Salicylic Acid, Jasmonic Acid, and Abscisic Acid Dependent Responses

To determine the impact on SA, JA and ABA regulation, we examined genes encoding hallmark enzymes comprising phenylalanine ammonia-lyase, *PAL* (for SA biosynthesis pathway), allene oxide synthase, *AOS*, and lipoxygenases, *LOX3* and *LOX10* (for JA biosynthesis pathway), and glycine-rich protein, *ABI* (for ABA biosynthesis pathway).

In roots, at early time-points (1 and 2 dai) upon infection of line CP888 with *F. verticillioides* (CP+FV), the expression level of *PAL* remained unchanged when compared to the mock control CP888 plants (CPC) (Figure 3). At 4 dai, a small downregulation of *PAL* was observed (Log_2_FC = −0.8, *p* = 0.04), and later the gene expression returned to the basal level at 8 dai. For line NK7328, similar results were obtained at 1 and 2 dai when infected with *F. verticillioides*. However, a small induction of *PAL* was observed at 4 dai (2.6-fold, *p* = 0.07) and was then modulated back to the basal level as in the mock control NK7328 plants (Figure 3).

In leaves, at 1 dai, there was no difference in the expression level of *PAL* between the *F. verticillioides* infected and mock control CP888 plants. A slight down-regulation of *PAL* was observed at 2 dai (Log_2_FC = −0.9, *p* = 0.02). At later time-points, *PAL* was expressed at the basal level. For plants of line NK7328, although the infection did not induce *PAL* at early time-points, this resulted in a significant induction of *PAL* at 8 dai (3.9-fold, *p* = 0.01) in comparison with the mock control NK7328 plants (Figure 3).

In terms of the JA pathway, in roots, at 1 dai, the expression levels of *LOX3, LOX10* and *AOS* were similar to the mock CP888 plants. At 2 dai, *LOX10* and *AOS* were significantly downregulated with Log_2_FC values of respectively −1.8 (*p* = 0.01), and −1.4 (*p* = 0.01). At later time-points, all of these three genes were expressed at basal levels. By contrast, for the *F. verticillioides* infected NK7328 plants, a small but significant induction of *LOX3* and *AOS* was observed at 2 dai, and then they expressed at the basal levels as in the control NK7328 plants (Figure 3).

In leaves at 1 dai, a downregulation of *AOS* was observed in the infected CP888 plants (Log_2_FC =−2.2, *p* = 0.02). A downregulation of *LOX10* (Log_2_FC = −1.3, *p* = 0.01), and *AOS* (Log_2_FC =−1.2, *p* = 0.01) were found at 2 dai. These two genes were regulated back to the basal levels at later time-points. Conversely, for the NK7328 plants at early infection stages, the expression levels of these three genes remained unchanged compared to the mock plants. The only significantly strong induction of *AOS* was observed in leaves of this cultivar at 8 dai (15.4-fold, *p* = 0.01) (Figure 3).

Meanwhile, for ABA pathway-mediated responses, in roots at 2 dai, a downregulation of *ABI* appeared in the infected CP888 plants (Log_2_FC = −1.8, *p* = 0.01). For the NK7328 plants, the expression level of *ABI* was unchanged at early time-points when compared to the control NK7328 plants. A small induction of *ABI* appeared at 8 dai (2-fold, *p* = 0.01).

Similar expression patterns of *ABI* were observed in leaves of each cultivar (Figure 3). In the infected CP888 plants, *ABI* was downregulated at early time-points, particularly at 1 dai (Log_2_FC = −2.0, *p* = 0.01) before regulating at the basal level. Nonetheless, when NK7328 plants were infected with the pathogen, a small induction of *ABI* was observed at all time-points except for 2 dai, displaying at 1 dai (1.9-fold, *p* = 0.02), 4 dai (2.2-fold, *p* = 0.01), and 8 dai (3.3-fold, *p* = 0.04).

In conclusion, data on the *PAL, LOX3*, *LOX10*, *AOS*, and *ABI* profiles indicate that *F. verticillioides* infection might result in the downregulation of SA, JA, and ABA biosynthesis pathways in the susceptible line CP888. By contrast, a small upregulation of these pathways was observed in the line *Bt/GT* NK7328. Thus, the SA, JA, and ABA-mediated resistance responses were likely to be activated stronger in plants of line NK7328 compared to line CP888 upon infection by *F. verticillioides*.

#### 3.3.2. Transcriptional Regulation of Downstream Pathogenesis-Related Proteins

To uncover the action on the transcriptional regulation of PRs, the expression of three genes, *PR2* (encoding β-1,3-glucanase), *PR3* (encoding chitinase) and *PR10* (encoding PR10 protein) downstream of the archetypal defense hormones were examined.

In roots, at 1 dai, a strong upregulation of *PR2* (23.6-fold, *p* = 0.01) and *PR3* (7.9-fold, *p* = 0.01) was observed in the CP888 plants when infected with *F. verticillioides* (Figure 3). From 2 dai onwards, both *PR2* and *PR3* were regulated back to the basal level with the exception of an upregulation of *PR2* at 8 dai (5.5-fold, *p* = 0.01). For NK7328 plants, upon infection by *F. verticillioides*, a concomitant upregulation of *PR2, PR3*, and *PR10* was observed at all time-points. For example, at 2 dai, there was a significantly strong induction of *PR2* (107.9-fold, *p* = 0.01), *PR3* (17-8-fold, *p* = 0.01), and *PR10* (5.5-fold, *p* = 0.01) in the infected NK7328 plants.

In leaves, upregulation of *PR2, PR3,* and *PR10* was shown in the infected CP888 plants at all time-points. At 2 dai, *PR2* increased 47.1-fold (*p* = 0.01) and *PR3* increased 20.1-fold (*p* = 0.01) versus the control CP888 plants. Meanwhile, for line NK7328, these genes were also strongly induced upon infection by *F. verticillioides* in which a significant upregulation of *PR10* was observed from 1 dai onwards, of *PR3* from 2 dai onwards, and of *PR2* from 4 dai onwards. Exceptionally, *PR2* was significantly down-regulated at early time-points (Figure 3).

In conclusion, data on the expression levels of *PR2*, *PR3*, and *PR10* indicated that the infection of *F. verticillioides* could result in a stronger upregulation of PRs production by line NK7328 when compared to line CP888.

#### 3.3.3. Transcriptional Regulation of Benzoxazinoids

When it comes to the action on the BXs biosynthesis pathways, we assessed three biomarker genes, *BX6, BX8* and *BX9,* encoding three respective hallmark enzymes, 2-oxoglutarate-dependent oxygenase 6, 2-oxoglutarate-dependent oxygenase 8, and 2-oxoglutarate-dependent oxygenase 9, in the BXs pathways.

In roots, upon infection by *F. verticillioides* at 2 dai, a small upregulation of *BX9* was shown at line CP888 (1.9-fold, *p* = 0.02). For line NK7328 plants, at 1 dai, *BX9* was downregulated (Log_2_FC = −1.2, *p* = 0.023). *BX6* (1.6-fold, *p* = 0.02) and BX8 (2.7-fold, *p* = 0.01) were slightly induced at 2 dai.

In leaves, for line CP888 at 1 dai, the infection resulted in a significant down-regulation of *BX9* (Log_2_FC = −0.9, *p* = 0.067) compared to the control CP888 plants. Meanwhile, for line NK7328 plants, at 1 dai, *BX6* (1.8-fold, *p* = 0.01) and *BX8* (2.5-fold, *p* = 0.07) were concomitantly induced. *BX8* remained upregulated afterwards, particularly at 8 dai (27-fold, *p* = 0.01). A small but significant regulation of BX6 was also observed at 8 dai (3.3-fold, *p* = 0.01).

In summary, upon infection by *F. verticillioides,* the biosynthesis of BXs seemed to be activated more strongly in line NK7328 than in line CP888 by upregulating *BX6* and *BX8* in both roots and leaves.

## 4. Discussion

There is no local recommendation on the selection of FER-resistant maize lines in Vietnam. Hybrid lines of CP888 and *Bt/GT* NK7328 are predominant in the Vietnamese smallholder maize farms. Our previous reports have shown that the higher contamination of both maize fields and post-harvest maize by fumonisins was observed in hybrid line CP888 when comparing to line *Bt/GT* NK7328 [4,13]. This spurred the question whether line CP888 was more susceptible to *F. verticillioides*. To answer this question, we evaluated the susceptibility of these two locally-used cultivars to infection by 12 local isolates of *F. verticillioides* using a seed germination assay and an infection assay on maize seedlings.

Firstly, the seed germination assay indicated that hybrid line CP888 was more susceptible to *F. verticillioides* than line *Bt/GT* NK7328. In fact, seeds of hybrid line CP888 showed a higher infection level by all the fungal isolates through a significant reduction of seed germination rate (Figure 1B). Despite the fact that the *F. verticillioides* infection also resulted in significant negative impacts on the seed germination of line NK7328, the overall disease levels seemed to be less than in line CP888. Likewise, the higher susceptibility of line CP888 was confirmed in the *in-planta* assay (Figure 2) which showed high disease severity in roots and leaves.

However, the susceptibility of maize cultivars varied in each isolate of *F. verticillioides*. For example, in the germination assay, isolates F26.31, F26.2, F22.11, and F17.1 were highly aggressive in line NK7328, completely suppressing seed germination, while less aggressiveness was observed for isolates F02.11, F04.11, F14.1, and F01.12 (Figure 1B). The different virulence levels were also confirmed among isolates by the *in-planta* assay (Figure 2). Isolates F01.12 and F04.11 were more virulent than isolate F14.22 on line CP888 maize seedlings.

To better understand the mode of action in these two maize cultivars upon infection by *F. verticillioides* F01.12, we verified a profile of SA-, JA-, ABA-, BXs-, and PRs pathway-related genes. SA and JA-dependent responses play an essential role in plant-pathogen interactions [23]. In the present work, we found that a downregulation of *PAL, LOX10* and *AOS* could be associated with the higher susceptibility of line CP888 to *F. verticillioides*. (Figure 3). Wang et al. (2016) [24] reported that genes associated with JA and SA pathways were significantly upregulated in line *BT-1* resistant maize when infected with *F. verticillioides*. Moreover, Lanubile et al. (2014) [25] showed that a significant stronger induction of JA signaling pathway was observed in CO441 resistant line maize when compared to CO354 susceptible line maize, although SA signaling remained unchanged. Meanwhile, Battilani et al. (2018) [26] demonstrated that *LOX3* activation is a major susceptibility factor of maize against *F. verticillioides*. Therefore, the earlier and higher expression of *LOX3* may result in reduced resistance of line CP888 to the pathogen.

In addition, ABA phytohormone has been demonstrated to play a significant role in plant response to abiotic and biotic stress [27]. For example, ABA synthesizing pathway-related genes were strongly induced in *BT-1* resistant maize line upon *F. verticillioides* infection, suggesting that higher transcript levels of ABA-related genes might be linked to increased host resistance to the pathogen [24]. A similar result was observed in line *Bt/GT* NK7328 with an upregulation of *ABI*, while for the susceptible line CP888, a downregulation of ABI was shown when infected with *F. verticillioides*. Ton et al. (2009) [27] documented that ABA-dependent stomatal closure is likely to function as a defensive barrier against the penetration of pathogens into plants. A study by Nguyen et al. (2016) [28] showed that *Fusarium* species can penetrate the stomata of immature maize leaves, although it is not the main entrance of these fungal pathogens to maize plants [29]. The upregulation of *ABI* might be responsible for the stomatal closure in line NK7328. To summarize, these results provide evidence that the higher resistance of line *Bt/GT* NK7328 to *F. verticillioides* might be linked to the stronger induction of plant hormone biosynthesis pathways when compared to line CP888.

Regarding the involvement of PRs in the plant defense responses against *F. verticillioides*, we found that *F. verticillioides* infection resulted in a strong induction of genes encoding anti-fungal proteins: β-1,3-glucanase (*PR2*), chitinase (*PR3*), and *PR10* in seedlings of both cultivars. The higher transcript level of these genes might contribute to the increased resistance of line *Bt/GT* NK7328 when compared to line CP888. In agreement with Lanubile et al. (2012) [21], the higher expression levels of PRs encoding genes were found in kernels of the resistant line compared to kernels of the susceptible line upon infection by *F. verticillioides*. Campos-Bermudez et al. (2013) [17] have demonstrated that *F. verticillioides* infection was responsible for an upregulation of PRs encoding genes in maize plants. Collectively, these results suggest that stronger upregulation of PRs encoding genes might contribute to the higher resistance of line NK7328 against *F. verticillioides* compared to line CP888.

Furthermore, BXs and their derivatives, such as 2,4–dihydroxy–1,4–benzoxazin–3–one, 2,4–Dihydroxy–7–methoxy–2H–1,4–benzoxazin–3(4H)–one, and 6–methoxybenzoxazolin–3– one, are well-known as defense secondary metabolites in maize against fungal pathogens [30,31]. Despite the fact that biosynthesis pathways of BXs in maize have been well documented [20], roles of BXs in plant resistance responses against *F. verticillioides* remain poorly understood. In the present study, gene expression data showed that a stronger induction of *BX6* and *BX8* was observed in line NK7328 seedlings compared to line CP888, suggesting that the biosynthesis of BXs might be activated more strongly in line NK7328 than in line CP888 upon *F. verticillioides* infection. Ding et al. (2015) [32] demonstrated that the resistance response of maize to *Bipolaris maydis*, a fungal pathogen causing corn leaf blight, was associated with the upregulation of BXs synthesizing genes (e.g., *BX1* and *BX8*). Antimicrobial activity of BXs’ derivatives against *B. maydis* was also confirmed [32]. A BXs-dependent maize defense system has also been demonstrated to have involvement in responses to *Spodoptera littoralis,* a leaf feeder [33]. However, in some cases, *F. verticillioides* is capable of detoxifying BXs metabolites by activating gene clusters *FDB1* and *FDB2* [34]. While the detoxification of BXs does not link to its endophytic colonization to maize seedlings [35], it might result in an increase in fumonisin production by *F. verticillioides.* Baldwin et al. (2019) [19] demonstrated that the knockdown of BXs detoxification-related genes of *F. graminearum* results in a significant reduction of deoxynivalenol produced by this fungus in wheat kernels.

Last but not least, the findings revealed that a systemic resistance observed in both roots and leaves in the response to *F. verticillioides* was more synchronistic in line *Bt/GT* NK7328 seedlings than in CP888 seedlings. Accordingly, the synchronistic resistance response was confirmed in line *Bt/GT* NK7328 by the concomitant activation of the defense-related genes in both roots and leaves. However, information on signal transduction pathways in the defense response of maize to *F. verticillioides* infection remain largely unexplored. Murillo et al. (1999) [36] supposed that accumulation of PRs is intervened at a distance from the infection sites of *F. verticillioides*.

In conclusion, the aforementioned data support the higher susceptibility of line CP888 maize to *F. verticillioides* compared to *Bt/GT* NK7328-maize line. The findings could be added to local recommendations in the selection of FER resistant maize lines in which *Bt/GT* NK7328-maize line could be superior against local *F. verticillioides* isolates.

## 5. Conclusions

In present study, the virulence variability of *F. verticillioides* population residing in maize fields of Vietnamese central highlands was reported. Most importantly, the findings evidenced that a higher resistance of line *Bt/GT* NK7328 to *F. verticillioides* might be associated with a stronger induction of phytohormones (e.g., SA, JA, and ABA), pathogenesis-related proteins (e.g., PR10 protein, glucanase and chitinase), and BXs synthesizing-related genes. On the other hand, downregulation of the phytohormones encoding genes coupled with lower transcriptional expression levels of genes related to biosynthesis pathways of PRs and BXs, might result in the increased susceptibility of line CP888 upon *F. verticillioides* infection. Optimistically, our findings provide valuable information to understand the distinctive resistance mechanism of local maize genotypes against local *F. verticillioides* isolates, which could help in the selection of a *Fusarium* ear rot resistant maize line tailored for local use.

## Data Availability

The data presented in this study are available in the supplementary material.

## Supplementary Materials

The following are available online at www.mdpi.com/article/10.3390/jof7090724/s1, Figure S1: Working flow of the *in planta* assay, Table S1: A list of 12 *Fusarium verticillioides* isolates derived from Vietnamese central highlands’ maize fields between 2017 and 2019. Table S2: Primers used for DNA sequencing [37]. Table S3: Primers of target genes and reference genes used in this study [38,39,40].
