# Supplementary material for: Molecular Insights into Defense Responses of Vietnamese Maize Varieties to Fusarium verticillioides Isolates
Source: J Fungi (Basel). 2021 Sep 4;7(9):724. doi: 10.3390/jof7090724 (PMC8469167; doi:10.3390/jof7090724)
Supplement: Supplementary file 1 [file jof-07-00724-s001.zip › jof-1348578-supplementary.pdf]

Table S1. A list of 12 *Fusarium verticillioides* isolates derived from Vietnamese central highlands' maize fields between 2017 and 2019.

			Accession number		
**Isolate**	**Host**	**Location**	***TEF-1α***	***RBP1***	***RBP2***
F01-12	Maize kernel	Dak Lak	MZ559332	MZ615354	MZ559290
F02-11	Maize kernel	Dak Lak	MZ559333	MZ615355	MZ559291
F11-12	Maize kernel	Dak Lak	MZ359353	NA	MZ559311
F12-2	Maize kernel	Dak Lak	MZ559355	MZ615375	MZ559313
F17-1	Maize kernel	Dak Lak	MZ559361	MZ615381	MZ559319
F22-11	Maize kernel	Dak Lak	MZ559367	MZ615387	MZ559325
F04-11	Maize kernel	Dak Nong	MZ559341	MZ615362	MZ559299
F06-12	Maize kernel	Dak Nong	MZ559345	MZ615366	MZ559303
F14-1	Maize kernel	Dak Nong	MZ559357	MZ615377	MZ559315
F14-22	Maize kernel	Dak Nong	MZ559359	MZ615379	MZ559317
F26-2	Maize kernel	Dak Nong	MZ559370	MZ615390	MZ559328
F26-31	Maize kernel	Dak Nong	MZ559371	MZ615391	MZ559329

Translation elongation factor 1 alpha gene (*TEF-1α*), *RBP1* (RNA polymerase largest subunit), *RBP2* (RNA polymerase second largest subunit). NA: not applicable.

Table S2. Primers used for DNA sequencing. Adapted from O'Donnell et al. 2010 [37].

Locus	Primers	Sequence (5′-3′)
EF1-α (Translation elongation factor 1α)	EF1	ATGGGTAAGGARGACAAGAC
	EF2	GGARGTACCAGTSATCATG
RBP1 (RNA polymerase largest subunit)	F7	CRACACAGAAGAGTTTGAAGG
	R9	TCARGCCCATGCGAGAGTTGTC
RBP2 (RNA polymerase second largest subunit)	5f2	GGGGWGAYCAGAAGAAGGC
	7cr	CCCATRGCTTGYTTRCCCAT

Table S3. Primers of target genes and reference genes used in this study.

Gene	Forward primer	Reverse primer	Reference
PAL	AAGAAGGTGAACGAGCTGGA	GTTGTCGTTCACGGAGTTGA	Tzin et al. 2017 [38]
LOX3	CGTGTAAACGGGAAGAGAGC	CCAATGATTGCAACAAGCAC	
LOX10	GACATCCTCTCGTCGCACTC	ATGAACCCCTCGATCTCCTT	
BX6	GAGTGCATGCAGAACCTGAA	GCAGGAGGATGGTGAAGAAG	
PR10	GTCATGCCGTTCAGCTTCAT	TGTTCTTGCACTCGACTTG	Ding et al. 2015 [32]
AOS	ACCTGTTCACGGGCACCTAC	CGAGGAGCGAGGAGAAGTTG	
BX8	GATACCTGCCGGTGAGAGAG	GGGAACGTGTGGAAGATGAG	
BX9	GCAACATGAGGTACGTGTGC	GCAGCGATCTTGAATTCCTT	
PR2	GCGCAGACCTACAACCAGA	GGAGAAATTGATGGGGTACG	Lanubile et al. 2010 [39]
PR3	GGCTCTACGCCTACGTCAAC	GATGGAGAGGAGCACCTTGA	
ABI	GCGAGATCCTCGACTCCAAG	GGGCTTGGTTAACGGTGATG	Ding et al. 2015 [32]
EF-1α	TGGGCCTACTGGTCTTACTACTGA	ACATACCCACGCTTCAGATCCT	Lin et al. 2014 [40]
β-TUB	CTACCTCACGGCATCTGCTATGT	GTCACACACACTCGACTTCACG	

Phenylalamine ammonia-lyase (putative) (*PAL*); Pathogenesis-related protein 10 (*PR10*); Lipoxygenase 3 (*LOX3*); Lipoxygenase 10 (*LOX10*); 2-oxoglutarate-dependent oxygenase (*BX6*); UDP-glucosyltransferase (*BX8*/*BX9*); Allene oxide synthase (*AOS*); 1,3-β-glucanase (*PR2*); Chitinase (*PR3*); Homology to glycin-rich protein (*ABI*); Elongation factor 1 alpha (*EF-1α*); Beta tubulin (*β-TUB*).

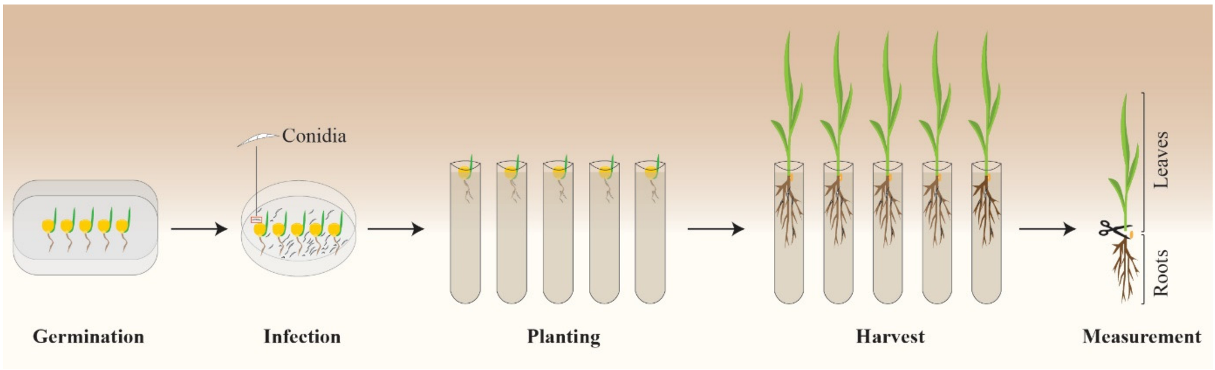

Figure S1. Working flow of the *in planta* assay
